# First nation-wide study of diabetic retinopathy in Poland in the years 2013–2017

**DOI:** 10.1007/s00592-020-01540-6

**Published:** 2020-06-04

**Authors:** Milena Kozioł, Michał S. Nowak, Monika Udziela, Paweł Piątkiewicz, Iwona Grabska-Liberek, Jacek P. Szaflik

**Affiliations:** 1 Department of Analyses and Strategies, Ministry of Health, 15 Miodowa str., 00-952 Warsaw, Poland; 2 Department of Ophthalmology, Medical University of Warsaw, Public Ophthalmic Clinical Hospital (SPKSO), 13 Sierakowskiego str., 03-709 Warsaw, Poland; 3 Provisus Eye Clinic, 112 Redzinska str., 42-209 Czestochowa, Poland; 4 Saint Family Hospital Medical Center, 19 Wigury str., 90-302 Lodz, Poland; 5 Department of Internal Diseases, Diabetology and Endocrinology, Medical University of Warsaw, 8 Kondratowicza str., 03-242 Warsaw, Poland; 6 Department of Ophthalmology, Centre of Postgraduate Medical Education, 231 Czerniakowska str., 01-416 Warsaw, Poland

**Keywords:** Diabetes mellitus, Diabetic retinopathy, Laser photocoagulation, Pars plana vitrectomy, Anti-VEGF and steroid injections

## Abstract

**Aims:**

To assess the prevalence and time trends of diabetic retinopathy (DR) in the overall population of Poland from 2013 to 2017 and diagnose the risk factors of occurring DR among patients with diabetes mellitus (DM).

**Methods:**

Data from all levels of healthcare services at public and private institutions recorded in the National Health Fund (NHF) database were evaluated. International Classification of Diseases codes (ICD-9 and ICD-10) and unique NHF codes were used to identify DM type 1 and type 2 patients, DR and treatment procedures including laser photocoagulation, pars plana vitrectomy (PPV), anti-VEGF and steroid intravitreal injections.

**Results:**

The overall registered prevalence of DR in the entire population of Poland was 0.81%. The mean prevalence of DR was 20.01% in the population with type 1 DM and 9.70% in the population with type 2 DM. In the study period, women represented 56.36% of all individuals registered with DR and 55.09% of all DM patients. In Poland, only 6.34% of all DM patients with DR received specific treatment with laser photocoagulation of the retina (82.32%), PPV (11.56%), anti-VEGF or steroid injections (5.15% and 0.97%, respectively). Cox regression hazard analysis showed that the risk of DR was associated with DM treatment only by GPs, female sex, coexisting systemic diseases and urban residence in both type 1 and type 2 DM.

**Conclusions:**

A 5-year retrospective analysis reveals the mean prevalence of DR in the population with type 1 and type 2 DM in Poland was rather low.

## Introduction

According to the latest report of Vision Loss Expert Group of the Global Burden of Disease Study, the crude prevalence (at all ages) of visual impairment and blindness caused by diabetic retinopathy (DR) increased significantly between the years 1990 and 2015 in respect to global population [1]. The number of people affected by blindness due to DR increased from 0.2 million to 0.4 million, however, by moderate to severe vision impairment increased from 1.4 million to 2.6 million. An increase in the age-standardized prevalence of blindness and visual impairment caused by DR was observed in the high income sub-regions, North Africa and Middle East. Meanwhile, in all regions of sub-Saharan Africa, Latin America, Oceania, Caribbean, Central and Eastern Europe the prevalence of blindness and visual impairment due to DR decreased [1]. DR is also the leading cause of preventable blindness in working-age adults; the potential risk of blindness in an individual with diabetes mellitus (DM) is 2.4 times higher than in an individual without diabetes [2, 3]. The number of people affected by DM is expected to rise from 382 million in the year 2013 to 592 million by the year 2035 [4]. Although many studies on DR comprising at least 10,000 participants have been performed recently both in Europe and worldwide [5–13], none of them was based on a national base. According to those studies, the prevalence of DR varied from 16.3% among patients with DM type 2 in Portugal to 48.4% among patients with DM type 1 in Great Britain [6, 7]. The aim of our study was to assess the prevalence and time trends of diabetic retinopathy in the overall population of Poland in the years 2013–2017. This study was a part of the project entitled “Maps of Healthcare Needs—Database of Systemic and Implementation Analyses,” which was co-financed by the European Union funds through the European Social Fund under the Operational Programme Knowledge Education Development [14, 15].

## Materials and methods

**Fig. 1:**
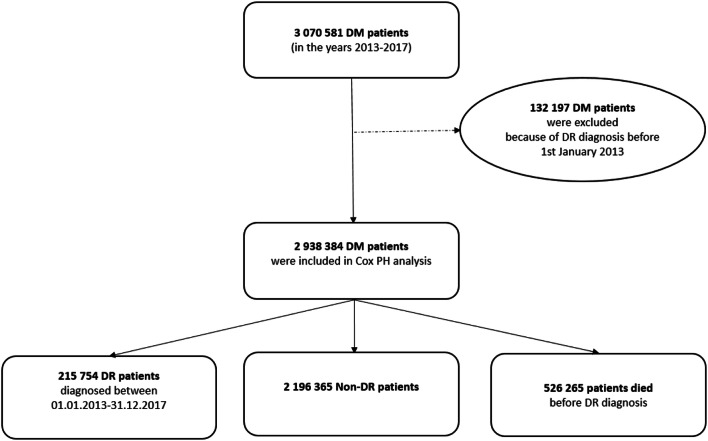
The study flowchart

In Poland, information related to all levels of healthcare services at public and private institutions financed from the public sources is recorded in the database of the National Health Fund (NHF). The NHF national database provides accurate population-based data and compiles both medical and socio-demographic data. The medical data include diagnoses coded according to the International Classification of Diseases, 10th Revision (in our NHF database only major ICD-10 codes without extensions are available), and all performed procedures coded according to the International Classification of Diseases, 9th Revision, the ICD-9 procedure codes and unique NHF codes corresponding to certain medical procedures. The socio-demographic data include the Polish personal identification number (PESEL), age, sex and place of residence of all patients. The NHF database also contains the records of all medications purchased by the Polish patients in pharmacies. The first part of the present study was a cross-sectional study. During the study period, in the years 2013–2017, each individual patient reported in NHF database with ICD-10 codes E10 and E11 was retrospectively identified by Polish personal identification number (PESEL) as having DM type 1 or type 2. If a patient had two different diagnoses, the more frequent ICD-10 code was taken into account. All diagnoses were then confirmed if the patient purchased antidiabetic drugs (and/or insulin) during the period of the study. Patients with the ICD-10 codes E12, E13 and E14 were excluded from the analysis. Following that, all patients reported in NHF database with the ICD-10 codes H36.0, H36.8 and H35.8 were also retrospectively identified by Polish personal identification number (PESEL) as having DR. The ICD-9 codes 14.24 and 14.25 were used to identify laser photocoagulation of the retina. The ICD-9 codes 14.73, 14.74, 14.75 and NHF codes B16, B17, B82 and B83 were used to identify the vitrectomy procedures. The ICD-9 code 14.762 and NHF codes B84 and B98 were used to identify anti-VEGF injections and, finally, the ICD-9 code 14.763 was used to identify steroid injections in all DR patients. The population data of Poland were obtained from the Central Statistical Office of Poland [16]. The number of all individuals with DR as well as the number of patients with type 1 and type 2 DM were collected. The overall prevalence of DR in Poland was calculated by dividing the total number of individuals with DR registered from 2013 to 2017 (minus patients lost to follow-up due to death) by the mid-2017 population of Poland. The mean prevalence of DR among type 1 and type 2 DM patients was calculated by dividing the number of individuals with the relevant DR code by the total number of patients with type 1 and type 2 diabetes, respectively. The point prevalence was calculated by dividing the number of individuals with DR by the number of patients with type 1 and type 2 DM on 31 December of each year of the study. The demographic characteristics of patients with DR were presented with the mean and standard deviation (the socio-demographic data including age, sex and place of residence were recorded anonymously). The statistical analysis also included the characteristics of DR treatment in Poland (with laser photocoagulation, anti-VEGF and steroid injections and vitrectomy). The second part of the present study was a retrospective population-based analysis. Cox proportional hazards regression (HRs) was used to evaluate the risk of DR in all patients between January 2013 and December 2017. The beginning of follow-up was defined as the first diagnosis of DM patients who were included to the study in the years 2013–2017. In particular, if patient was confirmed (the algorithm from the first part of the study) as DM patient in the years 2013–2017 and reported earlier in the system with E10 or E11 code, the first appearance has been considered. The system was validated from the year 2010. All DM with DR diagnoses made prior 01.01.2013 have been excluded from the study (right censoring). Follow-up time ended at the earliest date of first diagnosis of DR (outcome), with death (left censoring), or on 31 December 2017 (left censoring). HRs were calculated separately for individuals with type 1 and 2 DM (*ρ* values < 0.05 were considered statistically significant), and were adjusted for age, gender, place of residence, DM treatment conducted only by a General Practitioner (GP) and systemic diseases—hypertension and hypercholesterolemia. In order to identify the patients with hypertension and hypercholesterolemia the ICD10 codes reported during any consultation or hospitalizations in the years 2013–2017 have been considered. The codes for the particular diseases are included in “Appendix.” The value of Cramer’s V has been calculated in order to detect the multicollinearity of the explanatory variables. The goodness of the models has been validated with two measures: Cox–Snell Pseudo-*R*^2^ and concordance. R statistical software V. 3.5.2 was used for all analyses. The study flowchart is presented in Fig. 1. The present study adhered to the tenets of the Declaration of Helsinki for research involving human subjects. However, we did not need to obtain ethic committee approval, as the study protocol was approved by the Polish Ministry of Health, which is entitled by the law of the Republic of Poland to process the National Health Fund data.

## Results

**Fig. 2:**
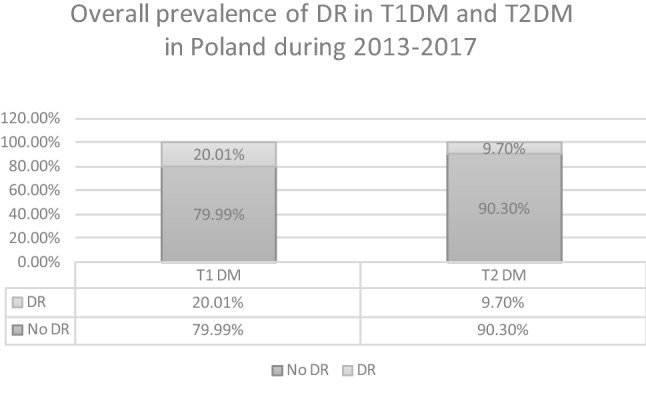
Overall prevalence of diabetic retinopathy in Type 1 and Type 2 DM patients in Poland in the period 2013–2017

**Fig. 3:**
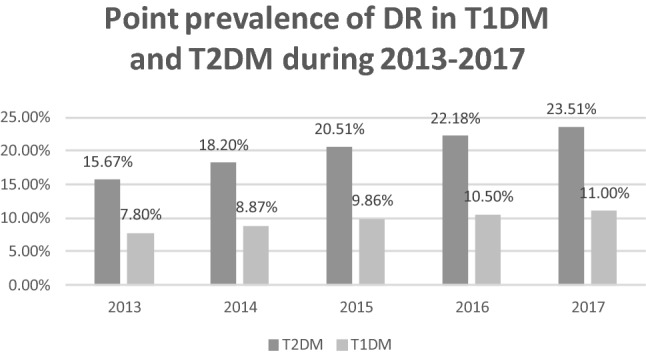
Point prevalence of diabetic retinopathy in Type 1 and Type 2 DM patients in Poland in the years 2013–2017

**Fig. 4:**
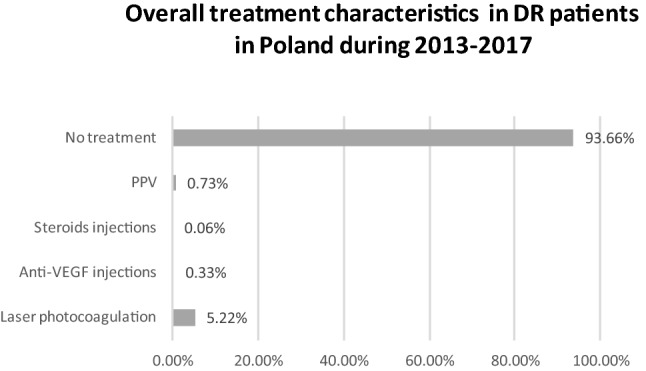
Characteristics of DR treatment in Poland in the years 2013–2017

During the study period, the total number of individuals with DM in Poland increased from 2,086,522 in the year 2013 to 2,635,249 in the year 2017. In total, 310,815 individuals with DR were diagnosed in the final year of the study (Table 1), after the exclusion of patients lost to follow-up due to death. The mid-2017 population of Poland was 38,422,346, according to the Central Statistical Office of Poland [16]. The overall registered prevalence of DR in the entire population of Poland was 0.81%. The mean prevalence of DR was 20.01% in the population with type 1 DM and 9.70% in the population with type 2 DM (Fig. 2), and increased from 15.67% and 7.8% in the year 2013 to 23.51% and 11.0% in the year 2017, respectively (Fig. 3). Demographic and clinical characteristics of DR and DM patients in Poland in the years 2013–2017 are presented in Tables 1 and 2. In the study period, women represented 56.36% of all individuals registered with DR and 55.09% of all DM patients. In Poland, the majority of DR and DM patients in the years 2013–2017 lived or had lived in urban areas. Among the baseline study subjects in the year 2013, type 1 DM was diagnosed in 8.23% of DM patients and in 15.28% of individuals with DR. In 2017, the final year of the study, type 1 DM was diagnosed in 6.5% of DM patients and in 12.94% of individuals with DR. Type 2 DM was diagnosed in 91.77% of DM patients and in 84.72% of individuals with DR in the year 2013, and in 93.5% of DM patients and in 87.06% individuals with DR in the year 2017, respectively. The most common systemic disorder recognized in more than 90% of DR individuals was systemic hypertension. Characteristics of the treatment of diabetic retinopathy in Poland in the years 2013–2017 are presented in Table 3 and Fig. 4. During the study period, only 6.34% of DM patients with DR received treatment with laser photocoagulation of the retina, anti-VEGF or steroid injections and pars plana vitrectomy (PPV). 93.66% of individuals with DR did not receive any treatment. Among those who were treated, 82.32% received laser photocoagulation of the retina, 11.56% were treated with PPV, 5.15% received anti-VEGF injections and only 0.97% received steroid injections. In the years 2013–2017 the number of laser photocoagulations remained stable. However, the total number of PPVs increased by 30%—from 1641 in the year 2013 to 2135 in the year 2017, the rate of PPV used in DR treatment decreased from 0.93% in the 2013 to 0.69% in the year 2017. The number of anti-VEGF treatments and steroid injections increased by 330% and 74%—from 420 and 127 in the year 2013 to 1386 and 222 in the year 2017, respectively. However, the total number of DM patients with DR, treated with any method increased from 14,896 in the year 2013 to 16,442 in the year 2017, the rate of DM patients treated decreased from 8.0% in the year 2013 to 4.9% in the year 2017. Building the Cox PH regression models has been started with validating the collinearity of explanatory variables. The value of Cramer’s V for each variable in both models revealed to be less than 0.41 so we reject the hypothesis of collinearity.

**Table 1:** Total number of registered individuals with diabetic retinopathy, type 1 and type 2 diabetes in Poland from 2013 to 2017 (minus patients lost to follow-up due to death)

	2013 (*n*: %)	2014 (*n*: %)	2015 (*n*: %)	2016 (*n*: %)	2017 (*n*: %)
DR cases in type 1 DM patients	26,908	31,525	35,514	38,258	40,231
Gender: % Women	(13,579; 50.46%)	(15,789; 50.08%)	(17,764; 50.02%)	(19,013; 49.7%)	(19,990; 49.69%)
Residence: % Urban	(18,395; 68.36%)	(21,431; 67.98%)	(24,021; 67.64%)	(25,719; 67.23%)	(26,953; 67%)
DR cases in type 2 DM patients	149,240	183,810	218,179	246,755	270,584
Gender: % Women	(86,412; 57.9%)	(106,159; 57.75%)	(125,234; 57.4%)	(141,112; 57.19%)	(154,394; 57.06%)
Residence: % Urban	(108,671; 72.82%)	(133,285; 72.51%)	(157,402; 72.14%)	(177,593; 71.97%)	(194,191; 71.77%)
Type 1 DM patients	171,689	173,249	173,195	172,551	171,152
Gender: % Women	(82,602; 48.11%)	(82,737; 47.76%)	(82,083; 47.39%)	(81,181; 47.05%)	(80,015; 46.75%)
Residence: % Urban	(109,834; 63.97%)	(110,597; 63.84%)	(110,189; 63.62%)	(109,418; 63.41%)	(108,295; 6.27%)
Type 2 DM patients	1,914,833	2,071,027	2,211,533	2,349,306	2,464,097
Gender: % Women	(1,073,429; 56.06%)	(1,156,889; 55.86%)	(1,230,798; 55.65%)	(1,304,525; 55.53%)	(1,366,188; 55.44%)
Residence: % Urban	(1,289,180; 67.33%)	(1,388,561; 67.05%)	(1,475,715; 66.73%)	(1,560,011; 66.4%)	(1,630,034; 66.5%)

**Table 2:** Demographic and clinical characteristics of patients with diabetic retinopathy in Poland

Characteristic	Baseline subjects seen in year 2013	All subjects seen in year 2017	Subjects lost to follow-up
Age (mean; SD)	65.61 ± 11.62 years	67.83 ± 11.59 years	73.82 ± 10.11 years
0–18 (*n*: %)	408 (0.23%)	708 (0.23%)	0 (0.00%)
19–39 (*n*: %)	4959 (2.82%)	7387 (2.38%)	141 (0.05%)
40–49 (*n*: %)	7604 (4.32%)	11,642 (3.75%)	484 (0.16%)
50–59 (*n*: %)	31,216 (17.72%)	37,526 (12.07%)	2406 (0.77%)
60–69 (*n*: %)	65,446 (37.15%)	112,514 (36.20%)	9535 (3.07%)
70 + (*n*: %)	66,515 (37.76%)	141,038 (45.38%)	24,570 (7.91%)
Women (*n*: %)	99,991 (56.77%)	174,384 (56.11%)	18,754 (6.03%)
Men (*n*: %)	76,157 (43.23%)	136,431 (43.89%)	18,382 (5.91%)
Urban residence	127,066 (72.14%)	221,144 (71.15%)	27,222 (8.76%)
Rural residence	49,082 (27.86%)	89,671 (28.85%)	9914 (3.19%)
Diabetes mellitus t. 1 (*n*: %)	26,908 (15.28%)	40,231 (12.94%)	5739 (1.85%)
Diabetes mellitus t. 2 (*n*: %)	149,240 (84.72%)	270,584 (87.06%)	31,397 (10.10%)
Systemic hypertension (*n*: %)	160,900 (91.34%)	280,162 (90.14%)	33,622 (10.82%)
Hypercholesterolemia (*n*: %)	9805 (5.57%)	17,527 (5.64%)	1248 (0.40%)

**Table 3:** Characteristics of diabetic retinopathy treatment in Poland in the years 2013–2017

Treatment methods	2013 *n* (%)	2014 *n* (%)	2015 *n* (%)	2016 *n* (%)	2017 *n* (%)
Laser photocoagulation	12,708 (7.21%)	13,109 (6.09%)	13,544 (5.34%)	13,011 (4.57%)	12,699 (4.09%)
Anti-VEGF injections	420 (0.24%)	525 (0.24%)	684 (0.27%)	1060 (0.37%)	1386 (0.45%)
Steroid injections	127 (0.07%)	145 (0.07%)	131 (0.05%)	140 (0.05%)	222 (0.07%)
Pars plana vitrectomy (PPV)	1641 (0.93%)	1657 (0.77%)	1805 (0.71%)	1895 (0.66%)	2135 (0.69%)
No treatment	162,077 (92.01%)	200,761 (93.23%)	238,438 (93.99%)	269,936 (94.71%)	295,557 (95.09%)
No. of all diabetic retinopathy patients	176,148 (100%)	215,335 (100%)	253,693 (100%)	285,013 (100%)	310,815 (100%)

Among the DM patients in Poland, the analysis of Cox proportional hazards regression (Tables 4, 5) showed that the treatment of DM by a GP was a significant risk factor for the occurrence of any DR, especially in subjects with type 1 diabetes (HR 2.59). HR was also higher for women and urban residents. In comparison with patients aged 50–59, HR of developing DR was 12% higher in patients with type 2 DM aged 60–69 and in all other age groups the ratio was reduced. The highest risk of developing DR among patients with type 1 DM was observed in the age group of 50–59 years, and it was reduced in all other age groups. Both systemic hypertension and hypercholesterolemia increased HR to over 40% in type 1 DM, and they also turned out to be very important factors in type 2 DM, HR 29% and 42%, respectively. The concordance of DM type 1 model and DM type 2 model was of value 0.68 and 0.61, relatively. Cox-Snell Pseudo-*R*^2^ of DM type 1 model and DM type 2 model was of value 0.13 and 0.11. The final models also demonstrated the incidence of DR stratified by each variable. In subjects with type 1 DM, the incidence of DR increased from 110.9 events per 10,000 person-years in age group 0–18 years to 320.2 events per 10,000 person-years in age group 50–59 years. In subjects with type 2 DM, the incidence of DR increased from 48.4 events per 10,000 person-years in age group 0–18 years to 172.6 events per 10,000 person-years in age group 60–69 years. In both type 1 and type 2 DM populations, the incidence of DR was higher in women versus men, 243.0 events per 10,000 person-years versus 237.7 events per 10,000 person-years and 145.7 events per 10,000 person-years versus 143.6 events per 10,000 person-years, respectively.

**Table 4:** Cox proportional hazards for diabetic retinopathy risk factors among patients with type 1 DM in Poland

Variables	Type 1 DM patients HR, 95% CI, *p* value	Number of observations in the group	DR cases; % of the group	DR cases per 10,000 person-years	Number of DR cases between *n*–*n* + 1 year since the inclusion into the study; % of all DR cases							
					0–1	1–2	2–3	3–4	4–5	5–6	6–7	7–8
Age												
0–18	0.38 (0.36–0.40) *p* < 0.001	21,190	1250; 5.9%	110.9	158; 12.6%	95; 7.6%	85; 6.8%	246; 19.6%	190; 15.2%	182; 14.56%	157; 12.56%	137; 10.96%
19–39	0.77 (0.75–0.79) *p* < 0.001	38,592	4689; 12.15%	207.2	229; 4.88%	155; 3.31%	249; 5.31%	1083; 23.1%	882; 18.81%	859; 18.32%	695; 14.82%	537; 11.45%
40–49	0.98 (0.95–1.01) *p* = 0.014	18,686	3145; 16.83%	294.1	160; 5.09%	108; 3.4%	201; 6.39%	705; 22.42%	633; 20.13%	551; 17.52%	461; 14.66%	326; 10.37%
50–59 (ref)	1.00 (1.00–1.00)	33,265	6213; 18.68%	320.2	257; 4.14%	190; 3.06%	401; 6.45%	1456; 23.43%	1287; 20.71%	1122; 18.06%	812; 13.07%	688; 11.07%
60–69	0.92 (0.90–0.95) *p* < 0.001	35,680	5988; 16.78%	301.3	294; 4.91%	192; 3.21%	339; 5.66%	1535; 25.63%	1189; 19.86%	1083; 18.09%	771; 12.88%	585; 9.77%
70 +	0.58 (0.57–0.60) *p* < 0.001	37,916	3145; 8.29%	182.7	91; 2.89%	80; 2.54%	226; 7.19%	1028; 32.69%	799; 25.41%	621; 19.75%	463; 14.72%	305; 9.7%
Sex: Men versus Women	0.90 (0.88–0.91) *p* < 0.001	98,662	12,929; 13.1%	237.7 versus 243.0	714; 5.52%	451; 3.49%	857; 6.63%	3135; 24.25%	2530; 19.57%	2214; 17.12%	1729; 13.37%	1299; 10.05%
Urban residence	1.22 (1.20–1.24) *p* < 0.001	116,475	16,524; 14.19%	252.8 versus 218.7	756; 4.58%	524; 3.17%	1002; 6.06%	4109; 24.87%	3316; 20.07%	2906; 17.59%	2207; 13.36%	1704; 10.31%
DM treatment only in GPs	2.59 (2.53–2.66) *p* < 0.001	13,391	3008; 22.46%	392.5 versus 240.7	214; 7.11%	157; 5.22%	197; 6.55%	1008; 33.51%	597; 19.85%	401; 13.33%	295; 9.81%	139; 4.62%
Systemic hypertension	1.46 (1.42–1.49) *p* < 0.001	117,310	18,568; 15.83%	272.9 versus 177.8	632; 3.4%	457; 2.46%	1063; 5.72%	4721; 25.43%	3856; 20.77%	3412; 18.38%	2521; 13.58%	1906; 10.26%
Hypercholesterolemia	1.49 (1.43–1.56) *p* < 0.001	6058	1222; 20.17%	344.2 versus 236.5	42; 3.44%	29; 2.37%	70; 5.73%	314; 25.7%	236; 19.31%	236; 19.31%	172; 14.08%	123; 10.07%

**Table 5:** Cox proportional hazards for diabetic retinopathy risk factors among patients with type 2 DM in Poland

Variables	Type 2 DM patients HR, 95% CI, *p* value	Number of observations in the group	DR cases; % of the group	DR cases per 10,000 person-years	Number of DR cases between *n*–*n* + 1 year since the inclusion into the study; % of all DR cases							
					0–1	1–2	2–3	3–4	4–5	5–6	6–7	7–8
Age												
0–18	0.29 (0.24–0.36) *p* < 0.001	4291	79; 1.84%	48.4	11; 13.92%	6; 7.59%	10; 12.66%	15; 18.99%	13; 16.46%	9; 11.39%	11; 13.92%	4; 5.06%
19–39	0.46 (0.44–0.47) *p* < 0.001	110,437	2953; 2.67%	71.7	562; 19.03%	300; 10.16%	275; 9.31%	459; 15.54%	450; 15.24%	396; 13.41%	297; 10.06%	214; 7.25%
40–49	0.83 (0.82–0.85) *p* < 0.001	193,467	11,531; 5.96%	131.3	1710; 14.83%	953; 8.26%	1037; 8.99%	2122; 18.4%	1850; 16.04%	1743; 15.12%	1225; 10.62%	891; 7.73%
50–59 (ref)	1.00 (1.00–1.00)	630,436	49,315; 7.82%	153.5	5079; 10.3%	3155; 6.4%	4225; 8.57%	9838; 19.95%	8815; 17.87%	7761; 15.74%	6033; 12.23%	4409; 8.94%
60–69	1.12 (1.11–1.13) *p* < 0.001	898,251	74,894; 8.34%	172.6	8214; 10.97%	5153; 6.88%	6501; 8.68%	15,654; 20.9%	13,012; 17.37%	11,620; 15.52%	8589; 11.47%	6151; 8.21%
70 +	0.83 (0.82–0.84) *p* < 0.001	916,173	52,084; 5.68%	119.8	4739; 9.1%	3355; 6.44%	4443; 8.53%	12,220; 23.46%	9838; 18.89%	7822; 15.02%	5901; 11.33%	3766; 7.23%
Sex: Men versus women	0.96 (0.96–0.97) *p* < 0.001	1,236,217	105,959; 8.57%	143.6 versus 145.7	9826; 9.27%	5922; 5.59%	7464; 7.04%	17,482; 16.5%	14,836; 14%	12,859; 12.14%	9745; 9.2%	6763; 6.38%
Urban residence	1.28 (1.27–1.29) *p* < 0.001	1,815,422	136,299; 7.51%	154.9 versus 123.7	14,047; 10.31%	9141; 6.71%	11,787; 8.65%	29,182; 21.41%	24,365; 17.88%	20,960; 15.38%	15,951; 11.7%	10,866; 7.97%
DM treatment only in GPs	1.18 (1.17–1.19) *p* < 0.001	986,345	50,369; 5.11%	150.2 versus 130.4	8028; 15.94%	4281; 8.5%	4681; 9.29%	11,563; 22.96%	8661; 17.2%	6617; 13.14%	4263; 8.46%	2275; 4.52%
Systemic hypertension	1.29 (1.27–1.31) *p* < 0.001	2,382,903	174,414; 7.32%	148.1 versus 114.6	17,180; 9.85%	11,445; 6.56%	14,959; 8.58%	37,390; 21.44%	31,483; 18.05%	27,228; 15.61%	20,463; 11.73%	14,266; 8.18%
Hypercholesterolemia	1.42 (1.39–1.44) *p* < 0.001	104,013	10,225; 9.83%	202.2 versus 142.2	1080; 10.56%	761; 7.44%	903; 8.83%	2130; 20.83%	1823; 17.83%	1546; 15.12%	1184; 11.58%	798; 7.8%

## Discussion

This study evaluates the characteristics and trends of the prevalence of diabetic retinopathy in the population with both type 1 and type 2 diabetes mellitus in Poland, in the years 2013–2017. Since it is based on the overall population of Poland, it is the first study in Europe and in the world to provide data concerning the prevalence of DR on such a scale. This study reported the rate of registered patients with DM and DR in the entire population of Poland on the level about 6.80% and 0.81%, respectively, in 2017. During the study period, the total number of individuals with DM in Poland increased by 26.3%. Our finding is in agreement with the estimation of World Health Organization which projected that the total number of people with DM will double from 2000 to 2030. With the increasing number of people with diabetes, the number of DR and vision-threatening DR, has been estimated to rise to 191.0 million and 56.3 million, respectively, by 2030 [17]. The mean prevalence of DR in Poland was 20.01% in the population with type 1 DM and 9.70% in the population with type 2 DM, and it increased significantly—from 15.67% and 7.8% in the year 2013 to 23.51% and 11.0% in the year 2017, respectively. Direct comparison between our results and the findings obtained in DR studies from other countries is limited due to the differences in study design. This is mainly due to ethnic populations, different standards of living, varying sample sizes and lack of uniformity regarding DR definitions and reporting in different countries. Those studies did not comprise also nation-wide populations. However, the prevalence of DR among subjects with type 2 DM in Poland was similar to the prevalence of DR found among subjects with type 2 DM in a rural population of South India [18]. Our results were also close to the results of the RETINODIAB study from Portugal, in which any DR was detected in 16.3% patients with type 2 DM aged 40 and above [6]. Portugal is also a middle-income country, with the gross domestic product per capita similar to that of Poland [19]. In other recently published studies, the prevalence of DR among type 2 DM patients ranged from 20.1% per over 64 thousand patients from German/Austrian Diabetes Prospective Documentation Initiative [8], through 21.0% in over 11 thousand patients from the greater Wellington region in New Zealand [13],] to 28.3% per over 7.7 million subjects in the Clinical Practice Research Datalink (CPRD) database in Great Britain [7]. The prevalence of DR among type 1 DM patients was higher and ranged from 13.4% in India [17], through 29.0% in the Wisconsin Epidemiologic Study of Diabetic Retinopathy (WESDR) from the USA [20] and 42.3% in the greater Wellington region in New Zealand [13], to 48.4% in the CPRD database in Great Britain [7]. The incidence of DR in Poland was the highest in age group 50–59 years in type 1 DM patients and in age group 60–69 years in type 2 DM patients but in both groups was much lower than found in CPRD database in Great Britain [7]. In Poland, 51.5% of all DM patients were women, and this rate was stable over the study period. However, the proportion of women was 57.5% in type 2 DM patients and was significantly higher than in type 1 DM (about 50.0%). It was associated with the fact that type 2 diabetes is more common among older people, the majority of whom in Poland are women. It is mainly attributable to the excess male death rate characteristic of the post-Soviet areas [21]. In our study, men had lower HR of developing any DR in comparison with women. Our results were in agreement with the study from Japan which showed that females exhibit a significantly higher prevalence of proliferative diabetic retinopathy (in type 2 DM) at baseline and that female gender is an independent risk factor for the development of DR [22]. By contrast, the results of the studies from Portugal, Germany/Austria, India, New Zealand and Great Britain (in T2DM) showed that men were at higher risk of developing DR [6–8, 13, 23]; however, the results of the studies from China, South Korea, Singapore and Great Britain (in T1DM) did not show gender-related differences [7, 12, 24, 25]. Those studies also revealed that DR was associated with longer duration of diabetes, higher mean glycosylated hemoglobin, albuminuria, vascular accidents, systemic hypertension and insulin therapy (in type 2 DM) [6–8, 11, 13, 23–25]. In our study, any DR was also significantly associated with the treatment of DM only by GPs, systemic diseases and urban residence in both type 1 and type 2 DM patients. According to the Central Statistical Office of Poland, about 60.5% of the Polish population live in urban areas [16]. It implies a meaningful difference in the prevalence rate with respect to the place of residence, especially among patients with DR and type 2 DM (72.2%). However, this characteristic could also be related to underdiagnosis of DR among patients living in the countryside because of lower access to screening. The treatment of DM only by GPs was the most significant risk factor for the occurrence of any DR in Poland, especially in subjects with type 1 diabetes (HR 2.59). This strong association shows the importance of the lack of regular diabetic treatment on higher levels of healthcare. Limited availability of diabetic specialists and ophthalmologists results in poor awareness of the significance of regular examinations in respect of diabetic retinopathy. Our results were in agreement with the results of other studies from Great Britain which showed regular DR screening had a major impact on visual impairment among DM subjects [26]. Those studies also showed poor awareness of the significance of regular examinations in respect of diabetic retinopathy resulted in the increased DR prevalence in patients with type 1 DM. However, this association was not confirmed in patients with type 2 DM [27]. The characteristics of DR treatment highlighted other problem as well. Local/grid laser photocoagulation of the retina, which is now reserved mostly for non-center-involving DR according to the current treatment guidelines, remains the gold standard in DR treatment in Poland [28]. While anti-vascular endothelial growth factor (anti-VEGF) and intraocular corticosteroid therapies have become the first-line treatment [26], they have been used in only 6% of the DR patients treated in Poland. During the study period, the total rate of DM patients with DR, treated with any method, decreased from 8.0% in the year 2013 to 4.9% in the year 2017. This could result from the increase in the number of patients with asymptomatic mild DR requiring no treatment. On the other hand, the increased number of PPVs from 2013 to 2017 could be caused either by a higher number of advanced cases with tractional retinal detachment, by a higher number of surgeons performing vitreoretinal surgery or by the diffusion of the indication of PPV for macular edema with a tractional component. Without some more precise information on the diagnosis, these results on the treatment are hard to interpret.

There are also other limitations to the current study. The major one is limited availability of diabetes specialists and ophthalmologist which may result in underestimation of the number of people with DM and DR in Poland. The most important strengths of the present study are: the population size, national recruitment and the impact of its findings on the public health. However, our results are specific to Poland and do not describe different healthcare systems in Eastern Europe. The results of epidemiological studies addressing the DR prevalence in type 1 and 2 DM differed worldwide. The limitation of all these studies is reporting data of a single population, thus reducing the external validity of the study.

In summary, our study showed the prevalence of DR in the overall population of Poland in the years 2013–2017 as well as the existing risk factors and treatment methods. This is the first nation-wide study of DR in Eastern Europe. During the study period the mean prevalence of DR was 20.01% in the population with type 1 DM and 9.70% in the population with type 2 DM. The treatment of DM only by GPs, especially in type 1 DM patients, turned out to be the crucial risk factor for developing DR. Additionally, laser photocoagulation of the retina remains the gold standard of DR treatment in Poland.

## Appendix: Systemic diseases definition (according to ICD10 classification, version 2016)

**Table:**

ICD10 code	Explanation	Systemic disease in the model
I10 (with extensions)	Essential (primary) hypertension	Hypertension
I11 (with extensions)	Hypertensive heart disease	
I12 (with extensions)	Hypertensive renal disease	
I13 (with extensions)	Hypertensive heart and renal disease	
I15 (with extensions)	Secondary hypertension	
E78.0	Pure Hypercholesterolemia	Hypercholesterolemia
